# Necessity of sentinel lymph node biopsy in ductal carcinoma in situ patients: a retrospective analysis

**DOI:** 10.1186/s12893-021-01170-x

**Published:** 2021-03-22

**Authors:** Young Duck Shin, Hyung-Min Lee, Young Jin Choi

**Affiliations:** 1 Department of Anesthesiology and Pain Medicine, Chungbuk National University Hospital, Chungbuk National University College of Medicine, Cheongju, Republic of Korea; 2 Department of Surgery, Chungbuk National University Hospital, Chungbuk National University College of Medicine, 1 Chungdae-ro, Seowon-gu, Cheongju-si, Chungcheongbuk-do 28644 Republic of Korea

**Keywords:** Core-needle biopsy, Ductal carcinoma in situ, Predictive factor, Sentinel lymph node biopsy, Upstaging

## Abstract

**Background:**

Sentinel lymph node biopsy (SLNB) is unnecessarily performed too often, owing to the high upstaging rates of ductal carcinoma in situ (DCIS). This study aimed to evaluate the upstaging rates of DCIS to invasive cancer, determine the prevalence of axillary lymph node metastasis, and identify the clinicopathological factors associated with upstaging and lymph node metastasis. We also examined surgical patterns among DCIS patients and determined whether SLNB guidelines were followed.

**Methods:**

We retrospectively analysed 307 consecutive DCIS patients diagnosed by preoperative biopsy in a single centre between 2014 and 2018. Data from clinical records, including imaging studies, axillary and breast surgery types, and pathology results from preoperative and postoperative biopsies, were extracted. Univariate analyses using Chi-square tests and multiple logistic regression analyses were used to analyse the data.

**Results:**

The rate of upstaging to invasive cancer was 19.2% (59/307). DCIS diagnosed by core-needle biopsy (odds ratio [OR]: 6.861, 95% confidence interval [CI]: 2.429–19.379), the presence of ultrasonic mass-forming lesions (OR: 2.782, 95% CI: 1.224–6.320), and progesterone receptor-negative status (OR: 3.156, 95% CI: 1.197–8.323) were found to be associated with upstaging. The rate of sentinel lymph node metastasis was only 1.9% (4/202), and all were total mastectomy patients diagnosed by core-needle biopsy. SLNB was performed in 37.2% of 145 breast-conserving surgery patients and 91.4% of 162 total mastectomy patients. Among the 202 patients who underwent SLNB, 145 (71.7%) without invasive cancer on final pathology had redundant SLNB. Two of 59 patients (3.4%) with disease upstaged to invasive cancer had inadequate primary staging of the axilla, as the rate seemed sufficiently small.

**Conclusions:**

In patients with a preoperative diagnosis of DCIS, although an unavoidable possibility of upstaging to invasive cancer exists, axillary metastasis is unlikely. Only 2.7% of patients with DCIS undergoing total mastectomy were found to have sentinel lymph node metastases. SLNB should not be performed in breast-conserving surgery patients and should be reserved only for total mastectomy patients diagnosed by core-needle biopsy.

## Background

The incidence of axillary lymph node metastasis in pure ductal carcinoma in situ (DCIS) is < 1%; therefore, in principle, pure DCIS patients do not need to undergo axillary surgery, yet unnecessary axillary surgeries are performed too often [1, 2]. Nevertheless, it is estimated that 13.3–37.9% of patients with a preoperative histological diagnosis of DCIS are upgraded to invasive carcinoma on final postoperative histological examination [3, 4]. Factors associated with upstaging include palpability, tumour size ≥ 5 cm, ultrasonic mass-forming lesions, Van Nuys Classification III, and tumours located in the upper-outer quadrant [5]. Individual surgeons or institutions may have different rationales for sentinel lymph node biopsy (SLNB) depending on the above-mentioned characteristics. They may proceed with SLNB during primary surgery to minimise the possibility of reoperation and missing true sentinel lymph nodes (SLNs) in the second procedure. SLNB is performed to reduce the complications caused by unnecessary axillary dissection (AD); however, it can also result in additional shoulder pain, sensory disturbance, lymphedema, and limited arm movement [6]. Therefore, SLNB must be used only where necessary to avoid over-treatment, which can cause unnecessary morbidity.

Guidelines recommend axillary evaluation based on the type of breast surgery in DCIS patients scheduled for curative surgery. According to the recommendations of the Korean Breast Cancer Society and National Comprehensive Cancer Network, patients diagnosed with DCIS do not need axillary surgery if they plan to undergo breast-conserving surgery (BCS); conversely, patients who plan to undergo total mastectomy (TM) are strongly recommended to undergo axillary evaluation using SLNB because additional SLNB cannot be performed after TM [7, 8]. In cases where DCIS patients undergo TM without SLNB, it would be prudent to perform axillary staging via AD if pathological examination reveals invasive cancer. In clinical practice, however, axillary evaluation, including SLNB, is frequently performed in DCIS patients undergoing BCS because of the possibility of upstaging to invasive cancer and avoiding a second operation.

In this study, we analysed the medical records of patients diagnosed with DCIS scheduled for BCS or TM. We aimed to evaluate the rate of upstaging to invasive cancer after surgery and identify the clinicopathological factors associated with upstaging. In addition, we evaluated the prevalence of axillary lymph node metastasis in preoperatively diagnosed DCIS patients and analysed factors related to axillary metastasis and their clinical role. We also assessed the rates of SLNB among DCIS patients based on breast surgery type and investigated clinical factors associated with its use.

## Methods

This study was conducted in accordance with the Declaration of Helsinki and was approved by the Institutional Review Board (IRB) of Chungbuk National University Hospital, Republic of Korea (approval number: CBNUH 201910012). The requirement for informed consent was waived by the IRB of Chungbuk National University Hospital, Republic of Korea owing to the retrospective nature of the study. The records of all patients who underwent surgery after a DCIS diagnosis by preoperative biopsy at our institution from January 2014 to December 2018 were retrospectively analysed.

The following variables were analysed: (1) patient demographics, (2) preoperative clinical characteristics, including estimated preoperative tumour size, palpability, tumour location, presence of mammographic calcification, and ultrasonographic mass, (3) preoperative biopsy method, including core-needle biopsy, vacuum-assisted breast biopsy (VABB), and excisional biopsy, and (4) type of breast surgery, including BCS or TM and axillary surgery (SLNB or AD). Permanent pathology results were evaluated to check for upstaging to invasive cancer and the presence of axillary lymph node metastasis. Pathological tumour size, including in situ and invasive cancer, comedo necrosis, nuclear grade, oestrogen receptor (ER) and progesterone receptor (PR) status, human epidermal growth factor receptor 2 (HER2) status, and Ki-67 status were evaluated. ER and PR statuses were determined by immunohistochemistry, and tumours with ≥ 1% of positively stained tumour cells were classified as positive. HER2 status was considered positive if immunohistochemistry was 3 + or fluorescence in situ hybridisation (HER2/neu to chromosome 17 ratio) was > 2.0. Proliferation activity was assessed by immunostaining with the Ki-67 antibody (Dako, Carpinteria, CA, USA). Ki-67 expression was scored as the percentage of positive tumour cells with any nuclear staining and recorded as the mean percentage of positive cells. The Ki-67 labelling index was evaluated by one pathologist, and the proportion of proliferating cells was determined by counting ≥ 500 tumour cells. A Ki-67 labelling index ≥ 14% was considered positive.

Baseline characteristics of the patients, including preoperative and postoperative characteristics, were evaluated using descriptive statistics. Univariate analysis using the Chi-square test was performed to analyse the factors predictive of and associated with invasive disease on final pathology and axillary lymph node metastasis. Multiple logistic regression analysis was performed to calculate the probability of invasive disease on final pathology using odds ratios (ORs) and 95% confidence intervals (CIs). According to the type of breast surgery, factors predictive of axillary surgery in DCIS patients were evaluated using the Chi-square test. Differences were considered statistically significant at *p*-values < 0.05. All statistical analyses were performed using SPSS statistical software, version 17.0 (SPSS Inc., Chicago, IL, USA).

## Results

A total of 307 patients were identified as having DCIS after core-needle biopsy (n = 174, 56.7%), VABB (n = 8, 2.6%), and excisional biopsy (n = 125, 40.7%) during the study period. The patients’ demographic, tumour, and treatment characteristics are presented in Table 1. The median age was 52 years. Most patients had a preoperative tumour size ≤ 5 cm (n = 279, 90.9%). Seventy-three (23.8%) patients presented with palpable lesions, and 192 (62.5%) presented with mammographic calcification. A total of 198 (64.5%) patients had ultrasonic mass-forming lesions, while 27 (8.8%) had multicentric tumours. Of these, 145 (47.2%) patients underwent BCS and 162 (52.8%) underwent TM. Primary SLNB was performed in 202 (65.8%) patients, of whom four (1.9%) with SLN metastasis underwent AD. There were no cases of AD without SLNB. Overall, axillary lymph node metastasis was identified in four (1.4%) of 307 patients. The rate of upstaging to invasive cancer was 19.2% (n = 59); 95% of patients had T1 lesions (n = 56), while no tumours were upstaged to T3/4 lesions (Table 1). One hundred and forty-five patients (47.2%) had comedo and 125 (40.7%) had cribriform as the main histological type of DCIS. Ninety (29.3%) patients had high nuclear grade, and half (49.8%) had comedo necrosis. Over two-thirds of the patients had endocrine-responsive tumours, and 74 (24.1%) had HER2-positive tumours. A high Ki-67 labelling index was reported in 126 (41.0%) patients (Table 1).

**Table 1:** Demographics, tumour characteristics, and treatment of patients with DCIS

Characteristic		Patients (n = 307) (%)
Age, years	Median (range)	52 (21–82)
	≤ 40	36 (11.7)
	> 40 and ≤ 60	201 (65.5)
	> 60	70 (22.8)
Preoperative characteristics		
Tumour size, cm	≤ 2	155 (50.5)
	> 2 and ≤ 5	124 (40.4)
	> 5	28 (9.1)
Palpability	Non-palpable	234 (76.2)
	Palpable	73 (23.8)
Mammographic calcification	Present	192 (62.5)
	Absent	115 (37.5)
Ultrasonic mass	Present	198 (64.5)
	Absent	109 (35.5)
Tumour location	Upper-outer	105 (34.2)
	Upper-inner	50 (16.3)
	Lower-outer	60 (19.5)
	Lower-inner	65 (21.2)
	Multicentric	27 (8.8)
Biopsy method	Core-needle biopsy	174 (56.7)
	VABB	8 (2.6)
	Excisional biopsy	125 (40.7)
Postoperative characteristics		
Type of breast surgery	BCS	145 (47.2)
	TM	162 (52.8)
Type of axillary surgery	None	105 (34.2)
	SLNB only	198 (64.5)
	SLNB and ALND	4 (1.3)
Upstaging to invasive cancer	Yes	59 (19.2)
	No	248 (80.8)
Axillary lymph node metastasis	Positive	4 (1.3)
	Negative	303 (98.7)
Permanent size of DCIS, cm	≤ 2	156 (50.8)
	2–5	124 (40.4)
	> 5	27 (8.8)
Permanent T stage	pTis	248 (80.8)
	pT1mic	5 (1.6)
	pT1a–pT1c	51 (16.6)
	pT2	3 (1.0)
Nuclear grade	Low	67 (21.8)
	Intermediate	150 (48.9)
	High	90 (29.3)
Comedo necrosis	Present	153 (49.8)
	Absent	154 (50.2)
ER	Positive	241 (78.5)
	Negative	66 (21.5)
PR	Positive	211 (68.7)
	Negative	96 (31.3)
HER2	Negative	233 (75.9)
	Positive	74 (24.1)
Ki-67	Low	181 (59.0)
	High	126 (41.0)
Main type of DCIS	Cribriform	125 (40.7)
	Comedo	145 (47.2)
	Solid	37 (12.1)

*BCS* breast-conserving surgery, *DCIS* ductal carcinoma in situ, *ER* oestrogen receptor, *HER2* human epidermal growth factor receptor 2, *PR* progesterone receptor, *SLNB* sentinel lymph node biopsy, *TM* total mastectomy, *VABB* vacuum-assisted breast biopsy

Table 2 shows the clinicopathological predictive factors of DCIS for upstaging to invasive cancer. It was observed that patients who underwent TM had a significantly higher upstaging rate than patients treated with BCS (n = 41, 25.3% *vs.* n = 18, 12.4%; *p* = 0.004). The univariate analysis revealed that factors such as preoperative tumour size > 5 cm, multicentric disease, and ultrasonic mass-forming lesions occurred more frequently in upstaged patients. There was no significant difference in the presence of palpable lesions or mammographic calcification. Furthermore, upstaged patients experienced comedo necrosis, ER and PR negativity, and high nuclear grade and Ki-67 labelling index more frequently. There was no significant difference in HER2 positivity between upstaged and non-upstaged patients. When grouped by diagnostic method, 53 (30.5%) of 174 patients diagnosed with core-needle biopsy were upstaged, while only five (4.0%) of 125 patients diagnosed with excisional biopsy were upstaged to invasive cancer (*p* < 0.001).

**Table 2:** Clinicopathological predictive factors for DCIS upstaging to invasive cancer and lymph node metastasis

Variable	Upstaging to invasive cancer (%)			Axillary lymph node metastasis (%)		
	Present	Absent	*p*-value	Present	Absent	*p*-value
Size of DCIS, cm			0.048			0.017
≤ 2	27 (17.3)	129 (82.7)		1 (0.6)	154 (99.4)	
> 2 and ≤ 5	22 (17.7)	102 (82.3)		1 (0.8)	123 (99.2)	
> 5	10 (37.3)	17 (63.0)		2 (7.1)	26 (92.9)	
Palpability			0.177			0.215
Non-palpable	41 (17.5)	193 (82.5)		2 (0.9)	232 (99.1)	
Palpable	18 (24.7)	55 (75.3)		2 (2.7)	71 (97.3)	
Mammographic calcification			0.127			0.604
Present	17 (14.8)	98 (85.2)		3 (1.6)	189 (98.4)	
Absent	42 (21.9)	150 (78.1)		1 (0.9)	114 (99.1)	
Ultrasonic mass-forming lesions			0.001			0.135
Present	49 (24.7)	149 (75.3)		4 (2.0)	194 (98.0)	
Absent	10 (9.2)	99 (90.8)		0 (0.0)	109 (100.0)	
Tumour location			0.046			0.240
Upper-outer	19 (18.1)	86 (81.9)		3 (2.9)	102 (97.1)	
Upper-inner	13 (26.0)	37 (74.0)		0 (0.0)	50 (100.0)	
Lower-outer	14 (23.3)	46 (76.7)		0 (0.0)	60 (100.0)	
Lower-inner	5 (7.7)	60 (92.3)		0 (0.0)	65 (100.0)	
Multicentric	8 (29.6)	19 (70.4)		1 (3.7)	26 (96.3)	
Biopsy method			< 0.001			0.212
Core-needle	53 (30.5)	121 (69.5)		4 (2.3)	170 (97.7)	
V ABB	1 (12.5)	7 (87.5)		0 (0.0)	8 (100.0)	
E xcisional	5 (4.0)	120 (96.0)		0 (0.0)	125 (100.0)	
Breast surgery			0.004			0.057
BCS	18 (12.4)	127 (87.6)		0 (0.0)	145 (100.0)	
Mastectomy	41 (25.3)	121 (74.7)		4 (2.5)	158 (97.5)	
Comedo necrosis			0.028			0.995
Absent	22 (14.3)	132 (85.7)		2 (1.3)	152 (98.7)	
Present	37 (24.2)	116 (75.8)		2 (1.3)	151 (98.7)	
Nuclear grade			0.021			0.478
Low	6 (9.0)	61 (91.0)		0 (0.0)	67 (100.0)	
Intermediate	29 (19.3)	121 (80.7)		2 (1.3)	148 (98.7)	
High	24 (26.7)	66 (73.3)		2 (2.2)	88 (97.8)	
ER			0.003			0.864
Positive	38 (15.8)	203 (84.2)		3 (1.2)	238 (98.8)	
Negative	21 (31.8)	45 (68.2)		1 (1.5)	65 (98.5)	
PR			< 0.001			0.416
Positive	28 (13.3)	183 (86.7)		2 (0.9)	209 (99.1)	
Negative	31 (32.3)	65 (67.7)		2 (2.1)	94 (97.9)	
HER2 neu			0.201			0.966
Negative	41 (17.6)	192 (82.4)		3 (1.3)	230 (98.7)	
Positive	18 (24.3)	56 (75.7)		1 (1.4)	73 (98.6)	
Ki-67			0.010			0.714
Low	26 (14.4)	155 (85.6)		2 (1.1)	179 (98.9)	
High	33 (26.2)	93 (73.8)		2 (1.6)	124 (98.4)	
Type of DCIS			0.027			0.748
Cribriform	18 (14.4)	107 (85.6)		2 (1.6)	123 (98.4)	
Solid	4 (10.8)	33 (89.2)		2 (1.4)	143 (98.6)	
Comedo	37 (25.5)	108 (74.5)		0 (0.0)	37 (100.0)	
Total	59 (19.2)	248 (80.8)		4 (1.3)	303 (98.7)	

*BCS* breast-conserving surgery, *DCIS* ductal carcinoma in situ, *ER* oestrogen receptor, *HER2* human epidermal growth factor receptor 2, *PR* progesterone receptor, *VABB* vacuum-assisted breast biopsy

SLN metastasis was identified in four (1.9%) of 202 SNLB patients, and all four in the TM group underwent additional AD at the time of their mastectomy. All four patients with SLN metastasis presented with ultrasonic mass-forming lesions, and the initial DCIS diagnosis was based on core-needle biopsy findings. The invasive tumour size of each of the four patients was 0.2, 1.1, 1.3, and 3 cm, respectively. Among the 59 patients with invasive cancer, four (6.7%) had SLN metastasis, while no SLN metastasis was observed in the non-invasive cancer group. As there were only four SLN metastasis cases, we could not identify statistically significant clinicopathological predictors of axillary lymph node metastasis in DCIS patients diagnosed preoperatively (Table 2).

In the multivariate analysis, core-needle biopsy-diagnosed DCIS (OR: 6.861, 95% CI: 2.429–19.379), the presence of ultrasonic mass-forming lesions (OR: 2.782, 95% CI: 1.224–6.320), and PR-negative status (OR: 3.156, 95% CI: 1.197–8.323) were associated with upstaging to invasive cancer (Table 3). Preoperative tumour size, high-grade DCIS, and histological type of DCIS were not associated with increased risk of upstaging in our multivariate analysis. In addition, we found no significant association between breast surgery type and upstaging (Table 3).

**Table 3:** Independent risk factors for upstaging to invasive cancer in DCIS patients

Characteristic	OR	95% CI	*p*-value
Preoperative tumour size > 5 cm	1.049	0.347–3.172	0.933
Presence of ultrasonic mass	2.782	1.224–6.320	0.015
Multicentric tumour location	1.244	0.374–4.138	0.722
Core-needle biopsy as a diagnostic method	6.861	2.429–19.379	< 0.001
TM	1.738	0.796–3.796	0.166
Comedo necrosis	0.972	0.406–2.327	0.950
High nuclear grade	1.263	0.321–4.970	0.738
ER-negative	0.830	0.290–2.378	0.729
PR-negative	3.156	1.197–8.323	0.020
High Ki-67 labelling index	1.167	0.519–2.623	0.710
Comedo type DCIS	0.972	0.406–2.327	0.950

*CI* confidence interval, *DCIS*ductal carcinoma in situ, *ER* oestrogen receptor, *OR* odds ratio, *PR* progesterone receptor, *TM* total mastectomy

Primary SLNB was performed in 54 (37.2%) patients treated with BCS and 148 (91.4%) treated with TM (Table 4). Fourteen patients did not undergo SLNB during TM. Of those, 10 were diagnosed with DCIS using excisional biopsy; they did not undergo SLNB at the surgeons’ discretion. The remaining four patients had a history of contralateral malignancy and were diagnosed with bilateral breast cancer. These patients refused axillary surgery, including SLNB, because they wanted to preserve their ipsilateral arm.

**Table 4:** Patients offered primary SLNB according to final pathology and breast surgery type

	BCS (n = 145)		TM (n = 162)	
	DCIS	Invasive cancer	DCIS	Invasive cancer
SLNB (n = 202)	38	16	107	41
No SLNB (n = 105)	89	2	14	0
	127 (87.6%)	18 (12.4%)	121 (74.7%)	41 (25.3%)

*BCS* breast-conserving surgery, *DCIS* ductal carcinoma in situ, *SLNB* sentinel lymph node biopsy, *TM* total mastectomy

SLN metastasis was identified in only four patients (2.7%) undergoing TM, and none was observed among patients undergoing BCS. Of the 59 patients with a final diagnosis of invasive cancer, two patients in the BCS group were not subjected to SLNB during primary surgery (undertreated group). However, they were subsequently offered SLNB as a secondary procedure, which revealed negative axilla. Final histology findings showed that none of the 14 patients in the TM group who were not offered SLNB initially had invasive cancer, whereas 55 (27.2%) of the 202 patients initially offered SLNB were found to have invasive cancer on final pathology, and 147 patients (72.8%) without invasive cancer on final pathology had redundant SLNB.

Table 5 shows the clinicopathological predictors for performing SLNB in patients with DCIS who underwent BCS. Among DCIS patients who underwent BCS, SLNB was performed more frequently in those with a large tumour size (*p* < 0.001), palpable lesions (*p* = 0.011), ultrasonic mass-forming lesions (*p* = 0.012), high nuclear grade (*p* = 0.001), comedo necrosis (*p* = 0.007), and core-needle biopsy-diagnosed DCIS (*p* < 0.001).

**Table 5:** Clinicopathological predictors of SLNB in DCIS patients undergoing BCS

Characteristic	BCS (n = 145) (%)		
	SLNB (n = 54, 37.2%)	None (n = 91, 62.8%)	*p*-value
Preoperative tumour size, cm			< 0.001
≤ 2	27 (26.7)	74 (73.3)	
> 2 and ≤ 5	27 (61.4)	17 (38.6)	
> 5 cm	0 (0.0)	0 (0.0)	
Palpability			0.011
Non-palpable	36 (31.6)	78 (68.4)	
Palpable	18 (58.1)	13 (41.9)	
Mammographic calcification			0.608
Present	30 (39.5)	46 (60.5)	
Absent	24 (34.8)	45 (65.2)	
Ultrasonic mass-forming lesions			0.012
Present	42 (45.2)	51 (54.8)	
Absent	12 (23.1)	40 (76.9)	
Tumour location			0.071
Upper-outer	26 (45.6)	31 (54.3)	
Upper-inner	13 (61.9)	8 (38.1)	
Lower-outer	8 (30.8)	18 (69.2)	
Lower-inner	7 (17.1)	34 (82.9)	
Multicentric	0 (0.0)	0 (0.0)	
Biopsy method			< 0.001
Core-needle biopsy	47 (69.1)	21 (30.9)	
VABB	0 (0.0)	6 (100.0)	
Excisional biopsy	7 (9.9)	64 (90.1)	
Comedo necrosis			0.007
Absent	26 (28.6)	65 (71.4)	
Present	28 (51.9)	26 (48.1)	
Main type of DCIS			0.002
Cribriform	18 (25.0)	54 (75.0)	
Comedo	29 (55.8)	23 (44.2)	
Solid	7 (33.3)	14 (66.7)	
Nuclear grade			0.001
Low	7 (14.9)	40 (85.1)	
Intermediate	29 (46.8)	33 (53.2)	
High	18 (50.0)	18 (50.0)	

*BCS* breast-conserving surgery, *DCIS* ductal carcinoma in situ, *SLNB* sentinel lymph node biopsy, *VABB* vacuum-assisted breast biopsy

## Discussion

In this study, one-fifth of women with a preoperative diagnosis of DCIS had tumours upstaged to invasive cancer. Further, most patients without invasive cancer on final pathology had redundant SLNB, and few patients with disease upstaged to invasive cancer had inadequate primary staging of the axilla. Because it is impossible to eliminate under-treatment, the rate found in this study seems acceptable. The patients underwent successful SLNB after primary surgery.

The upstaging rate in this study is within the range of that suggested by previous reviews [9, 10], which showed a wide variation in the upstaging rate among patients with a preoperative diagnosis of DCIS, around 9–52% [9] and 10–38% [10]. This variation originated from various inclusion criteria and the biopsy method.

Many studies [5, 11–14] have attempted to identify risk factors for the upstaging of DCIS to invasive cancer. Factors associated with upstaging include palpability, tumour size ≥ 5 cm, a mass on imaging, young age at diagnosis, tumours located in the upper-outer quadrant, Van Nuys classification III, etc. The association between the presence of comedo necrosis or nuclear grade and upstaging has also been investigated, but the results are conflicting [12, 15–17]. In this study, core-needle biopsy-diagnosed DCIS, PR-negativity, and the presence of an ultrasonic mass were significantly associated with upstaging.

In South Korea, many DCIS patients with suspicious microcalcifications are diagnosed with surgical biopsy with wire localisation rather than VABB. One reason is that the cost of VABB is not covered by medical insurance, but surgical biopsy is. Although the number of cases diagnosed with VABB was relatively small, there was an obvious difference in the upstaging rate by biopsy method: 30.5% (n = 53) after core-needle biopsy, 12.5% (n = 1) after VABB, and 4% (n = 5) after excisional biopsy. This is similar to previous studies [1, 16, 18–21], which showed relatively high upstaging rates for core-needle biopsy (13.6–36.0%), and upstaging rates of 7–24% and 13.5% for VABB and excisional biopsy, respectively. VABB and excisional biopsy can obtain more representative tissue specimens than core-needle biopsy, which may explain why they were associated with lower upstaging rates than core-needle biopsy.

In this study, comedo necrosis, DCIS with high nuclear grade, and ER-negative status were significant factors for upstaging in the univariate analysis, but not in the multivariate analysis. Instead, PR-negative DCIS remained an independent risk factor for upstaging in the multivariate analysis. Although studies on hormonal receptor status as a predictor for upstaging are rare, our finding that PR-negative DCIS is an independent predictor for invasive cancer is in agreement with other studies [5, 22, 23].

We revealed that DCIS presenting as an ultrasonic mass was an independent predictor for upstaging to invasive cancer. This finding agrees with other studies suggesting that 56% of DCIS presents as a sonographic mass upstaged on final pathology. Szynglarewicz et al*.* [24] concluded that even a small ultrasonic mass indicates a high risk of upstaging invasive cancer after curative surgery. This radiological finding reflects the higher potential of local invasiveness, suggesting that cancer penetrated into the basement membrane of the duct and invaded deeper tissues.

Four patients identified as having SLN metastasis on frozen biopsy underwent AD during primary surgery. They also had invasive cancer on permanent pathology. Among patients who were upstaged to invasive cancer, 6.7% had SLN metastasis. In contrast, no SLN metastasis was seen in the non-invasive cancer group. This finding is consistent with previous studies [16, 23, 25, 26] that showed a low risk of axillary involvement (1.4–6%) in patients with preoperatively diagnosed DCIS. Because there were only four cases of SLN metastasis in this study, we could not identify statistically significant clinicopathological predictors for SLN metastasis in preoperatively diagnosed DCIS patients. According to the results of a nationwide study from the Danish Breast Cancer group [25], palpable DCIS, larger areas of DCIS, and younger age were associated with SLN metastasis. Additionally, several studies [1, 25] have shown high positive SLN rates after surgical excisional biopsy, indicating iatrogenic tumour cell displacement, although its clinical significance is questionable. However, in our study, among patients diagnosed with excisional biopsy, two-fifths underwent SLNB, and no patient was confirmed to have SLN metastasis.

Although guidelines do not recommend SLNB for planned BCS, over one-third of BCS patients underwent SLNB, and they were all negative for SLN metastasis. In this study, the factors associated with performing SLNB in BCS patients were large tumour size, palpable lesions, ultrasonic mass-forming lesions, high-grade DCIS, and comedo necrosis after core-needle biopsy. These were also the factors associated with upstaging in several previous studies [5, 11–17]. In addition, only 12.4% of BCS patients were identified as having invasive cancer on permanent pathology with no SLN metastasis confirmation. Therefore, to reduce unnecessary axillary surgery complications, for DCIS patients who undergo BCS, it is unnecessary to implement SLNB as a primary surgery; instead, it is necessary to follow the guidelines carefully. In cases of DCIS diagnosed with excisional biopsy, it is rare for TM specimens to show upstaging. For selective DCIS patients diagnosed with excisional biopsy, SLNB is also likely to be omitted, even when TM is performed, if there is no gross residual disease.

This study has several limitations. First, this was a retrospective study and may have included selection bias. Second, this study included a small number of patients in a single institution; thus, it is difficult to generalize the findings of the study in all patient populations. A multi-institutional prospective study is needed. Finally, we could not identify the clinical significance of upstaging and SLN metastasis in DCIS patients because of the short follow-up period.

## Conclusions

This study demonstrated that DCIS diagnosed with core-needle biopsy, PR-negative status, and ultrasonic mass-forming lesions were significantly associated with upstaging to invasive cancer. While the risk of upstaging was 19.2%, the rate of SLN metastasis was very low. Additionally, only a few DCIS patients undergoing TM were found to have SLN metastases. Thus, SLNB should not be performed in BCS patients. Rather, SLNB should be reserved only for patients undergoing TM. In carefully selected DCIS patients diagnosed with excisional biopsy, SLNB may be omitted, even during TM.

## Data Availability

The datasets used and/or analysed during the current study are available from the corresponding author on reasonable request.

## Abbreviations

AD: Axillary dissection
BCS: Breast-conserving surgery
CI: Confidence interval
DCIS: Ductal carcinoma in situ
ER: Oestrogen receptor
HER2: Human epidermal growth factor receptor 2
OR: Odds ratio
PR: Progesterone receptor
SLN: Sentinel lymph node
SLNB: Sentinel lymph node biopsy
TM: Total mastectomy
VABB: Vacuum-assisted breast biopsy

## References

[1] Francis AM, Haugen CE, Grimes LM, Crow JR, Yi M, Mittendorf EA (2015). Is sentinel lymph node dissection warranted for patients with a diagnosis of ductal carcinoma in situ?. Ann Surg Oncol.

[2] Intra M, Rotmensz N, Veronesi P, Colleoni M, Iodice S, Paganelli G (2008). Sentinel node biopsy is not a standard procedure in ductal carcinoma in situ of the breast: the experience of the European Institute of Oncology on 854 patients in 10 years. Ann Surg.

[3] Polom K, Murawa D, Wasiewicz J, Nowakowski W, Murawa P (2009). The role of sentinel node biopsy in ductal carcinoma in situ of the breast. Eur J Surg Oncol.

[4] Doyle B, Al-Mudhaffer M, Kennedy MM, O’Doherty A, Flanagan F, McDermott EW (2009). Sentinel lymph node biopsy in patients with a needle core biopsy diagnosis of ductal carcinoma in situ: is it justified?. J Clin Pathol.

[5] Lee SK, Yang JH, Woo SY, Lee JE, Nam SJ (2013). Nomogram for predicting invasion in patients with a preoperative diagnosis of ductal carcinoma in situ of the breast. Br J Surg.

[6] Veronesi U, Paganelli G, Viale G, Luini A, Zurrida S, Galimberti V (2003). A randomized comparison of sentinel-node biopsy with routine axillary dissection in breast cancer. N Engl J Med.

[7] Korean Breast Cancer Society. The 8th Korean Clinical Practice Guideline for Breast Cancer. 2019. http://www.kbcs.or.kr. Accessed 20 Apr 2019.

[8] National Comprehensive Cancer Network. Breast cancer—v.5. 2020. Accessed July 2020.

[9] van Deurzen CHM, Hobbelink MGG, van Hillegersberg R, van Diest PJ (2007). Is there an indication for sentinel node biopsy in patients with ductal carcinoma in situ of the breast? A review. Eur J Cancer.

[10] Kanbayashi C, Iwata H (2017). Current approach and future perspective for ductal carcinoma in situ of the breast. Jpn J Clin Oncol.

[11] Osako T, Iwase T, Ushijima M, Horii R, Fukami Y, Kimura K (2014). Incidence and prediction of invasive disease and nodal metastasis in preoperatively diagnosed ductal carcinoma in situ. Cancer Sci.

[12] Huo L, Sneige N, Hunt KK, Albarracin CT, Lopez A, Resetkova E (2006). Predictors of invasion in patients with core-needle biopsy-diagnosed ductal carcinoma in situ and recommendations for a selective approach to sentinel lymph node biopsy in ductal carcinoma in situ. Cancer.

[13] Han JS, Molberg KH, Sarode V (2011). Predictors of invasion and axillary lymph node metastasis in patients with a core biopsy diagnosis of ductal carcinoma in situ: an analysis of 255 cases. Breast J.

[14] Park AY, Gweon HM, Son EJ, Yoo M, Kim JA, Youk JH (2014). Ductal carcinoma in situ diagnosed at US-guided 14-gauge core-needle biopsy for breast mass: preoperative predictors of invasive breast cancer. Eur J Radiol.

[15] Wilkie C, White L, Dupont E, Cantor A, Cox CE (2005). An update of sentinel lymph node mapping in patients with ductal carcinoma in situ. Am J Surg.

[16] Yen TW, Hunt KK, Ross MI, Mirza NQ, Babiera GV, Meric-Bernstam F (2005). Predictors of invasive breast cancer in patients with an initial diagnosis of ductal carcinoma in situ: a guide to selective use of sentinel lymph node biopsy in management of ductal carcinoma in situ. J Am Coll Surg.

[17] Dillon MF, McDermott EW, Quinn CM, O’Doherty A, O’Higgins N, Hill AD (2006). Predictors of invasive disease in breast cancer when core biopsy demonstrates DCIS only. J Surg Oncol.

[18] Mannu GS, Groen EJ, Wang Z, Schaapveld M, Lips EH, Chung M (2019). Reliability of preoperative breast biopsies showing ductal carcinoma in situ and implications for non-operative treatment: a cohort study. Breast Cancer Res Treat.

[19] Prendeville S, Ryan C, Feeley L, O’Connell F, Browne TJ, O’Sullivan MJ (2015). Sentinel lymph node biopsy is not warranted following a core needle biopsy diagnosis of ductal carcinoma in situ (DCIS) of the breast. Breast.

[20] van Leeuwen RJH, Kortmann B, Rijna H (2020). Ductal carcinoma in situ after core needle biopsy: in which cases is a sentinel node biopsy necessary?. Breast Care (Basel).

[21] Grimm LJ, Ryser MD, Partridge AH, Thompson AM, Thomas JS, Wesseling J (2017). Surgical upstaging rates for vacuum assisted biopsy proven DCIS: implications for active surveillance trials. Ann Surg Oncol.

[22] Miller ME, Kyrillos A, Yao K, Kantor O, Tseng J, Winchester DJ (2016). Utilization of axillary surgery for patients with ductal carcinoma in situ: a report from the National Cancer Data Base. Ann Surg Oncol.

[23] Sato Y, Kinoshita T, Suzuki J, Jimbo K, Asaga S, Hojo T (2016). Preoperatively diagnosed ductal carcinoma in situ: risk prediction of invasion and effects on axillary management. Breast Cancer.

[24] Szynglarewicz B, Kasprzak P, Halon A, Matkowski R (2015). Preoperatively diagnosed ductal cancers in situ of the breast presenting as even small masses are of high risk for the invasive cancer foci in postoperative specimen. World J Surg Oncol.

[25] Holm-Rasmussen EV, Jensen MB, Balslev E, Kroman N, Tvedskov TF (2018). Risk factors of sentinel and non-sentinel lymph node metastases in patients with ductal carcinoma in situ of the breast: a nationwide study. Breast.

[26] Leonard GD, Swain SM (2004). Ductal carcinoma in situ, complexities and challenges. J Natl Cancer Inst.

